# Stable isotopes as tracers of trophic interactions in marine mutualistic symbioses

**DOI:** 10.1002/ece3.4712

**Published:** 2018-12-12

**Authors:** Christine Ferrier‐Pagès, Miguel Costa Leal

**Affiliations:** ^1^ Centre Scientifique de Monaco Monaco Monaco; ^2^ MARE – Marine and Environmental Sciences Centre Faculdade de Ciências da Universidade de Lisboa Lisbon Portugal

**Keywords:** compound‐specific stable isotope analysis, marine symbioses, mixing models, δ^13^C, δ^15^N

## Abstract

Mutualistic nutritional symbioses are widespread in marine ecosystems. They involve the association of a host organism (algae, protists, or marine invertebrates) with symbiotic microorganisms, such as bacteria, cyanobacteria, or dinoflagellates. Nutritional interactions between the partners are difficult to identify in symbioses because they only occur in intact associations. Stable isotope analysis (SIA) has proven to be a useful tool to highlight original nutrient sources and to trace nutrients acquired by and exchanged between the different partners of the association. However, although SIA has been extensively applied to study different marine symbiotic associations, there is no review taking into account of the different types of symbiotic associations, how they have been studied via SIA, methodological issues common among symbiotic associations, and solutions that can be transferred from one type of association with another. The present review aims to fill such gaps in the scientific literature by summarizing the current knowledge of how isotopes have been applied to key marine symbioses to unravel nutrient exchanges between partners, and by describing the difficulties in interpreting the isotopic signal. This review also focuses on the use of compound‐specific stable isotope analysis and on statistical advances to analyze stable isotope data. It also highlights the knowledge gaps that would benefit from future research.

## 1 INTRODUCTION

The term symbiosis or “the living together of different organisms” according to the original deBary definition (De Bary, 1879) is frequently used to describe a relationship in which all partners mutually benefit from the association. Later on, symbiosis has been described as a shifting continuum from mutualism to parasitism (Thrall, Hochberg, Burdon, & Bever, 2007). In mutualistic nutritional symbioses, both partners take advantage of being together by developing metabolic or nutritional interactions (Table 1). The exchange of nutrients allows the host and the symbionts to acquire nutrients that are limiting growth and reproduction, to expand their metabolic portfolio and, thus, the width and number of ecological niches to exploit, thereby avoiding competition with sympatric non‐symbiotic species.

**Table 1:** Nutritional benefits in mutualistic marine symbioses

Partner	Nutrient accessibility	Nutritional functions
Symbionts	Acquisition of inorganic nutrients (carbon, nitrogen) and dissolved organic matter (DOM) Access to carbon‐ and nitrogen‐rich host waste products Acquisition of diazotrophic‐derived nitrogen Acquisition or synthesis of essential metals and vitamins	Provision of entire symbiotic association by photosynthetically or chemosynthetically fixed carbon Transformation of inorganic nitrogen and/or N2 into organic nitrogenous compounds Provision of vitamins and metals to the entire association
Host	Acquisition of particulate and/or organic nutrients by capture of prey	Digestion and provision to the entire symbiotic association of essential carbon, nitrogen, and phosphorus compounds

**Figure 1:**
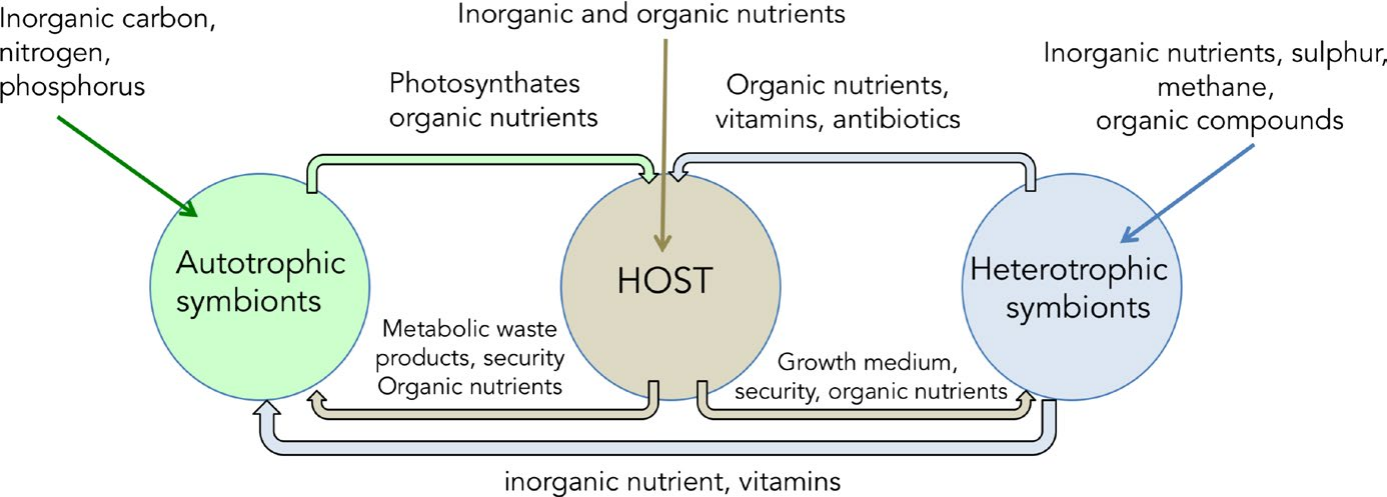
Nutritional relationships between an animal host and its phototrophic and heterotrophic symbionts

Mutualistic nutritional symbioses are widespread in nature. It is common to find plants that are associated with microorganisms that sequester nitrogen or phosphorus while receiving by‐products of host's photosynthesis (Lugtenberg, 2013; van Rhijn & Vanderleyden, 1995). In marine ecosystems, nutritional symbioses exist throughout the pelagic and benthic environments. They involve the association of a diverse range of algae, protists, sponges, sea squirts, corals, worms, and other marine invertebrates, with microorganisms such as bacteria (see reviews by Cavanaugh, 1994; Cavanaugh, McKiness, Newton, & Stewart, 2006; Petersen & Dubilier, 2009), cyanobacteria (reviewed in Carpenter & Foster, 2002), and dinoflagellates (see reviews by Goodson, Whitehead, & Douglas, 2001; Venn, Loram, & Douglas, 2008). In most associations (except for algae), the hosts are heterotrophic and prey on a wide range of particles to meet their nutritional demand. They, however, complement their nutrient intake by hosting symbionts, which provide them with carbon and other mineral sources in exchange for protection and access to catabolic products (Figure 1; Venn et al., 2008). In some cases, a fraction of the heterotrophically acquired nutrients is transferred from the host to the symbionts for their own needs (Tremblay, Gori, Maguer, Hoogenboom, & Ferrier‐Pagès, 2016). The amount and quality of nutrients exchanged between the partners can vary with environmental conditions (Baker, Freeman, Wong, Fogel, & Knowlton, 2018; Shantz, Lemoine, & Burkepile, 2016), as well as with the identity of the host and/or symbionts (Leal et al., 2015). Some host–symbiont associations can be species‐specific, with one host species being associated with a single symbiont species, as it is observed for some scleractinian corals that associate with particular *Symbiodinium* species (Abrego, Ulstrup, Willis, & Oppen, 2008; Baker, 2003). However, some invertebrates, such as sponges, can host extremely complex and diverse symbiont communities that are not strictly pairwise, or even endosymbiotic. Indeed, some marine sponges harbor complex communities of generalist symbionts that live associated with the host, but not necessarily within the host's cells (Erwin & Thacker, 2007). Nevertheless, there are also marine sponges hosting specific symbiont species (Wilkinson, Nowak, Austin, & Colwell, 1981). Even though some of the associations are species‐specific, the host may select best performing symbionts from a population of possible partners within the same species, a process known as partner choice (Akçay, 2017; Sachs, Mueller, Wilcox, & Bull, 2004). For example, scleractinian corals can be associated with different dinoflagellate clades of *Symbiodinium,* depending on the prevailing environmental conditions (Little, van Oppen, & Willis, 2004; Thornhill, Howells, Wham, Steury, & Santos, 2017). Overall, living with symbiotic partners and having access to different nutritional pathways is fundamental to many marine organisms, particularly those in nutrient‐poor environments.

Nutritional interactions between the partners are particularly difficult to identify in endosymbioses because they only occur in intact associations. Once the partners are isolated, their physiology changes completely. Additionally, exchanged nutrients are usually metabolites that cannot be tracked through visual observation of feeding behavior or gut contents, as often performed for addressing trophic interactions (Calado & Leal, 2015; Leal & Ferrier‐Pagès, 2016; Nielsen, Clare, Hayden, Brett, & Kratina, 2018). Nevertheless, our knowledge on nutritional exchanges in symbioses has benefited from recent methodological developments, such as proteomics, metabolomics, lipidomics, and isotopic biomarkers. Proteomics monitors change in protein expression of host and symbionts under different symbiotic states or environmental conditions, but requires genetic information on the studied models and advanced technology, such as liquid chromatography/electrospray ionization tandem mass spectrometry (Oakley et al., 2016). Metabolomics assess low molecular weight metabolite profiles (such as amino acids and lipids) of host and symbionts, and can detect fine‐scale changes in a rapid and quantitative manner (Hillyer, Tumanov, Villas‐Bôas, & Davy, 2016). These “omics” methods provide important information on the metabolic competences of each symbiotic partner. However, they are not very accurate for tracing metabolite exchanges or at evidencing the original nutrient source (Middelburg, 2014). In contrast, stable isotope analysis (SIA) provides information on the original nutrient source, which makes it one of the most helpful tools for assessing food web functioning, that is, tracing energy and nutrients from bottom producers to top predators, as well as for estimating trophic levels, resource use, and diet composition (Bouillon, Connolly, & Lee, 2008). In symbiotic associations, SIA can trace nutrients acquired by the host or the symbionts both from internal and external inorganic and organic sources, including exogenous inorganic nutrient sources (Davy, Allemand, & Weis, 2012; Ferrier‐Pagès et al., 2011).

SIA is based on biologically active elements existing in more than one isotopic form. Generally, the lighter isotope is more abundant in the environment than the heavier isotope, but their relative abundance is altered by biological, geochemical, and anthropogenic processes (Rundel, Ehleringer, & Nagy, 2012). These processes produce variations in the stable isotope ratio of constitutive molecules of plant and animal tissues, which provide a good record for the existence, and sometimes magnitude, of key processes involved with elemental cycling. For instance, the fractionation of nitrogen isotopes by consumers generates a gradient throughout the food web, with organisms in the bottom of the food chain displaying low δ^15^N values that gradually increase up through the food chain. This makes it possible to use this isotope to measure the trophic level of an individual. Information on such fractionation factor is, however, critical for using SIA to compare distributions of isotope ratios between the animal host and its symbionts or to the consumed food.

Numerous reviews have been published on the use of stable isotopes for coastal biogeochemistry (Bouillon et al., 2008), plant and animal ecology (Dawson, Mambelli, Plamboeck, Templer, & Tu, 2002; Martínez del Rio, Wolf, Carleton, & Gannes, 2009; Wolf, Carleton, & Martínez del Rio, 2009), and food web reconstructions (Boecklen, Yarnes, Cook, & James, 2011; Middelburg, 2014). However, an overarching review focusing on the use of SIA to study trophic interactions in marine symbiotic associations is still missing. In particular, there is still little information on how to interpret changes in isotopic signals of each symbiotic partner under different environmental and/or nutritional conditions, and how to use isotopes in natural abundance or in enrichment experiments tracing nutritional interactions in such complex associations. As previously stated, SIA has been applied to study different marine symbiotic associations, particularly chemosynthetic, photosynthetic, and nitrogen‐fixing symbioses. However, and despite sharing methodological and conceptual frameworks, there is no review that takes into account the different types of symbiotic associations, that addresses common issues, and that highlights similar solutions. As an increasing number of researchers are using SIA to study nutritional interactions in marine symbiotic associations, it is important to summarize the current knowledge, highlight the difficulties in using SIA in such symbiotic models, and provide novel and broad insights arising from such an overarching perspective among the various marine symbiotic associations. The present review aims to fill such gap in the scientific literature by: i) summarizing the current knowledge of how SIA have been applied to some key marine symbioses to unravel nutrient exchanges between partners, both under natural abundance or in enrichment experiments; and ii) describing the difficulties in interpreting the isotopic signal and using SIA in such associations. This review also focuses on the use of compound‐specific stable isotope analysis, statistical advances to analyze stable isotope data, as well as highlights the knowledge gaps that would benefit from future research.

## 2 MAIN MARINE SYMBIOSES STUDIED USING STABLE ISOTOPES

**Figure 2:**
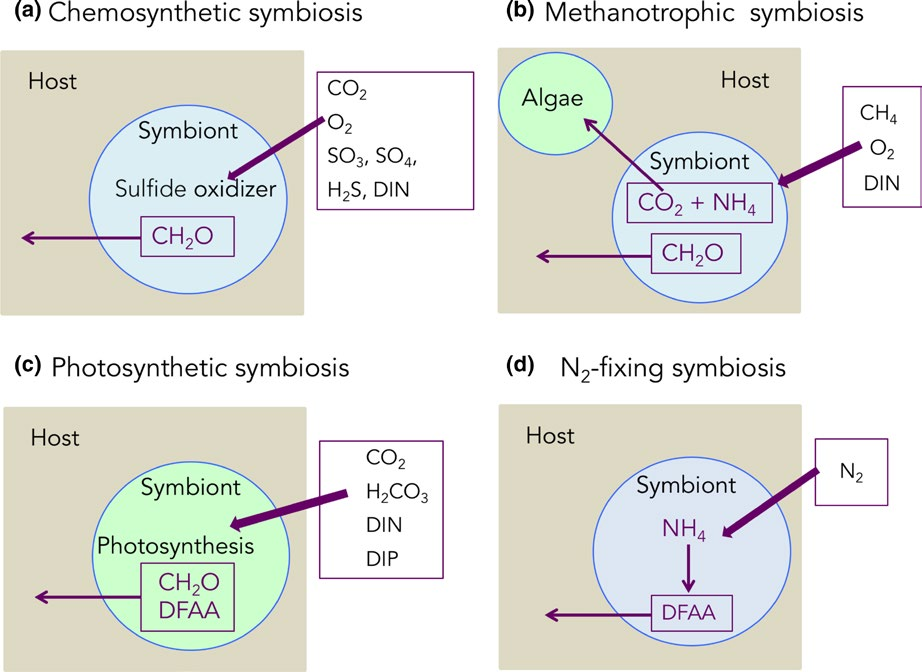
Main types of nutritional symbioses in the marine environment. (a) CO_2_, O_2_, SO_3_, SO_4_, H_2_S, DIN, CH_2_O: carbon dioxide, oxygen, sulfite, sulfate, hydrogen sulfide, dissolved inorganic nitrogen, saccharides; (b) CH_4_, NH_4_: methane and ammonium; (c) H2CO3, DIP, DFAA: bicarbonate, dissolved inorganic phosphorus, dissolved free amino acids, (d) N_2_: dinitrogen

SIA has been widely applied to study four main types of marine endosymbioses: chemosynthetic, photosynthetic, nitrogen‐fixing symbioses, and the heterotrophic bacteria–sponge type symbiosis (Figure 2). In all these relationships, the animal generally feeds on external particulate food sources such as phyto‐ and zooplankton or detrital organic matter and transfers a fraction of the heterotrophically acquired nutrients to the symbionts. Such transfer has been evidenced by several studies using prey labeled with the stable isotopes ^13^C and ^15^N and showing a transfer of isotopes from the host to the symbionts (Hughes, Grottoli, Pease, & Matsui, 2010; Piniak, Lipschultz, & McClelland, 2003; Tremblay, Maguer, Grover, & Ferrier‐Pagès, 2015). However, the type of nutrients that are transferred to the symbionts (e.g., sugars, lipids) is still an open question. The animal host also provides the symbionts with access to substrates (inorganic nutrients), which are necessary for their own generation of energy and biomass. In exchange, a portion of the inorganic nutrients fixed by the symbionts is transferred to the host for its own use (Figure 1).

First discovered at hydrothermal vents, ***chemosynthetic symbioses*** between certain invertebrates (sponges, snails, mussels, clams, nematodes, tube worms, shrimps, and sea urchins) and chemoautotrophic or methanotrophic bacteria are widespread in deep environments and are responsible for the high biomass observed in such extreme environments (reviewed in Cavanaugh et al., 2006). Chemosynthetic symbionts are primary producers even in the absence of light as they use a range of chemicals, such as reduced sulfur compounds, methane, and hydrogen as energy source to fix inorganic carbon or methane into organic molecules (Cavanaugh et al., 2006). Symbiotic bacteria mainly exchange with the host C1 to C3 carbon compounds, which are then used for host energy and biosynthesis. They are also able to take up ammonium or nitrate in their environment (Liao, Wankel, Wu, Cavanaugh, & Girguis, 2014) and even fix dinitrogen (Petersen et al., 2017).

***Photosynthetic symbioses*** are widespread associations involving cyanobacteria or microalgae symbionts. Cyanobacteria are mostly associated with marine sponges and diatoms (see reviews by Freeman & Thacker, 2011; Foster et al., 2011; Venn et al., 2008), although they are also symbionts of haptophytes or dinoflagellates (Not et al., 2016). *Prochloron*, a unicellular photosynthetic prokaryote, which is also part of the cyanobacteria phylum, is often associated with ascidians. Algae, such as dinoflagellates of the genus *Symbiodinium*, are associated with benthic animals such as reef‐building corals, sea anemones, tridacnid molluscs, jellyfish (*Cassiopea* sp.), and foraminifera (Freeman, Stoner, Easson, Matterson, & Baker, 2016; Sachs & Wilcox, 2006; Venn et al., 2008). In the pelagic environment, photosymbiotic interactions also exist between microalgae and other protists (radiolarian, foraminifera) or metazoans (ciliates, dinoflagellates), although the exact nature of this partnership is often not formally demonstrated. Overall, for all these photosynthetic symbioses, light is of prime importance because it is needed by the symbionts as an energy source to fix inorganic carbon into organic compounds called photosynthates, most of which are transferred to the host for its own use (Freeman et al., 2016). In addition, algal symbionts play a major role in the acquisition of inorganic nitrogen (ammonium, nitrate), phosphorus, and other macro‐ and micronutrients essential for the symbiosis (Tanaka, Miyajima, & Koike, 2006). Certain cyanobacterial symbionts, called diazotrophs, can also fix dinitrogen (N_2_) in a symbiotic association (see below).

The third type of symbiosis usually studied with SIA is the one developed between **animals or algae and diazotrophs**. In this association, diazotrophs (nitrogen‐fixing bacteria or cyanobacteria) provide their host with nitrogen, which is fixed through the reduction of N_2_ to NH_3_ using the nitrogenase complex. Diazotrophs are known to establish symbiosis with various invertebrates, such as sponges (Taylor, Radaz, Steger, & Wagner, 2007), annelid worms (Stat, 2016), corals (Bednarz, Grover, Maguer, Fine, & Ferrier‐Pagès, 2017; Lema, Willis, & Bourne, 2012), sea urchins (Guerinot & Patriquin, 1981), and protists such as dinoflagellates, diatoms, radiolarians, and tintinnids (Amin et al., 2015; Foster, Carpenter, & Bergman, 2006; Foster, Subramaniam, & Zehr, 2009).

The last well‐studied symbiosis is the one between **sponges and heterotrophic bacteria**. Many marine organisms harbor dense and diverse microbial communities, but the in situ activity and functions of these microbes are still poorly known, except in sponges. Concerning nutritional functions, stable isotope experiments have, among others, demonstrated a role of bacterial symbionts in the nitrogen cycle of sponges, in particular in nitrification, denitrification, and anaerobic ammonium oxidation (reviewed in Webster & Taylor, 2012). Genome sequencing also revealed that “Poribacteria” can undertake carbon fixation via the Wood–Ljungdahl pathway and provide the host with a source of vitamin B12 (Siegl et al., 2011).

## 3 STABLE ISOTOPES IN NATURAL ABUNDANCE FOR STUDYING NUTRITIONAL INTERACTIONS

Studies on natural stable isotope abundance are based on the small differences in isotopic ratios as found in nature (Hayes, 2001; Peterson & Fry, 1987). These changes in stable isotopic ratios are caused by the preferential use of the light isotopes compared to the heavy ones in many biological and chemical processes, which is called isotopic fractionation. SIA provides critical information on carbon and nitrogen origin, on the consumer's trophic ecology and trophic level, as well as on the ecology and evolution of predators and their trophic relationships (Bearhop, Adams, Waldron, Fuller, & MacLeod, 2004; Duarte, Flores, Vinagre, & Leal, 2017). For example, a specialized feeding strategy is evidenced by isotopic data that present low variability (or are homogeneous) among individuals, assuming that they are presented an isotopically consistent food source or mix of food sources. Conversely, large isotopic variability among individuals indicates that they specialize on different food sources or feed on isotopically distinct microhabitats in a heterogeneous “landscape”.

δ^13^C (^13^C:^12^C) and δ^15^N (^15^N:^14^N) are the two most common stable isotopes commonly used for assessing nutritional interactions. In the following sections, we describe these isotopes and their application to study nutritional interactions in marine mutualistic endosymbiosis. Other, less used stable isotopes, such as δ^18^O and δ^34^S, are also described.

### 3.1 δ^13^C isotopes

**Figure 3:**
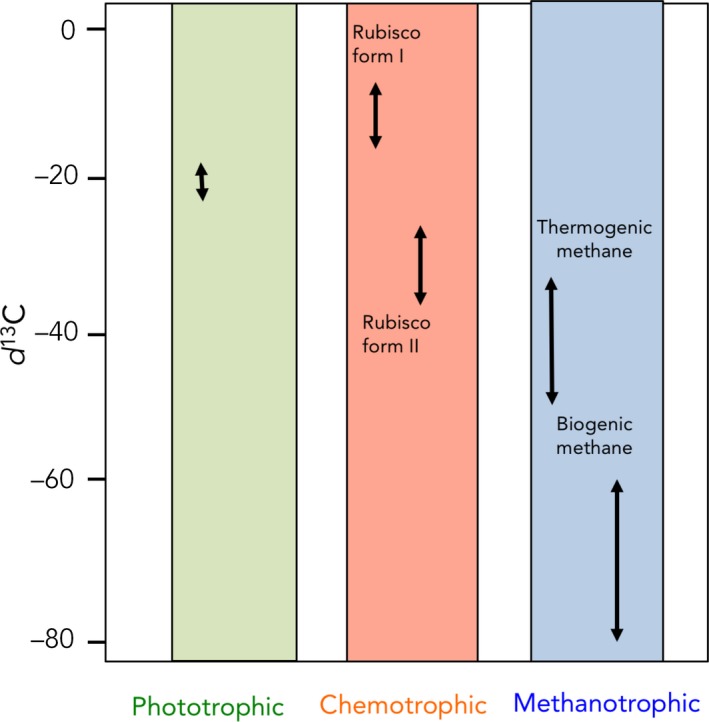
Different ranges of δ^13^C according to the C source

δ^13^C is a very useful tool to help distinguish autotrophic metabolisms, because enzymes involved in carbon fixation pathways discriminate differently against the use of the heavier carbon isotope (^13^C) (Cavanaugh et al., 2006). Consequently, different primary producer symbionts exhibit distinct δ^13^C values due to diverse carbon fixation pathways (Figure 3). For example, algae and phytoplankton preferentially assimilate the lighter isotope ^12^C and thus usually display δ^13^C values of −20‰ to −18‰ (Fry & Sherr, 1989; Gearing, Gearing, Rudnick, Requejo, & Hutchins, 1984; Goericke, 1994). δ^13^C derived from chemosynthesis occurring at vents is either considerably lighter than phytoplankton δ^13^C (enriched in ^12^C from −9‰ to −16‰) or heavier (depleted in ^12^C, from −27‰ to −35‰) (Fisher & Childress, 1992; Levin & Michener, 2002; Robinson et al., 2003). These two extreme chemosynthetic groups show contrasting δ^13^C values due to the use of two different forms of ribulose‐1,5‐bisphosphate carboxylase/oxygenase (Rubisco I and II), which catalyses the carbon fixation step of the Calvin‐Benson cycle. Rubisco form I discriminates more than Rubisco form II against ^13^C leading to lower, that is, more depleted, δ^13^C values (Robinson & Cavanaugh, 1995). Finally, symbioses involving methanotrophic bacteria can be even more depleted in ^13^C, with δ^13^C ranging from −37‰ to −55‰ for thermogenic methane, and from −60‰ to −80‰ for biogenic methane (Barry et al., 2002; Cavanaugh, 1993).

The δ^13^C result can be used as an indicator of the main food substrate(s) because it becomes enriched by only 0.4‰ to 1‰ between each trophic level (Conway, 1994; McCutchan, Lewis, Kendall, & McGrath, 2003). Therefore, in symbiotic associations, the δ^13^C values of host and symbionts should be similar. Comparisons between δ^13^C values of symbionts and host tissue, or between symbiont‐containing and symbiont‐free host tissues, can be theoretically used to trace the exchange of carbon between the symbionts and their animal host, and to estimate the importance of symbiont‐derived carbon supplied to the host. For example, in the coral–dinoflagellate symbiosis, in which carbon is mainly acquired through the dinoflagellates photosynthesis, the host tissue will display δ^13^C values in the same range as the symbionts (algal) values, that is, −11‰ to −16‰ (Alamaru, Loya, Brokovich, Yam, & Shemesh, 2009). In chemosynthetic symbioses, and similar to symbiotic corals, some animals can totally rely on the carbon transferred by the symbionts. This is the case of the solemyid protobranch *Solemya velum* that shows a δ^13^C value ranging from −38.4 to −45.3‰ (Conway & Capuzzo, 1991), the seep vestimentiferan tube worms and some methanotrophic mussels that show a δ^13^C value ranging from −40‰ to ‐ 65‰ (Kennicutt II et al., 1992), and thyasirid clams that show on average a δ^13^C value of −38‰ (Fiala‐Médioni, Boulegue, Ohta, Felbeck, & Mariotti, 1993). In some situations, however, the δ^13^C values of the host can be difficult to interpret, because host tissues rarely have δ^13^C values solely influenced by the symbionts. Most hosts can indeed use alternative (external) sources of nutrition, such as predation on free‐living zooplankton and phytoplankton, or uptake of dissolved organic material. Consequently, the δ^13^C value of the host tissue will be a mix of all the animal's diet. Such mixed diet is observed, for instance, in the western Pacific vent mussel *Bathymodiolus brevior*. In this species, the symbiont‐containing gill tissue showed δ^13^C values (−30.8‰ to −35.8‰) significantly lower than those from symbiont‐free foot tissue, demonstrating that this species supplemented its diet via filter feeding on external particles (Dubilier, Windoffer, & Giere, 1998). Some organisms also host several symbionts, such as the hydrothermal mussels *Bathymodiolus azoricus* that live in association with both thio‐ and methanotrophic bacteria, which ultimately confounds the δ^13^C value of the host (Cavanaugh, Wirsen, & Jannasch, 1992; Trask & Van Dover, 1999). For instance, small‐sized individuals can display δ^13^C values ranging from −27‰ to −34‰, suggesting that thiotrophy is the dominant nutritional pathway, with methanotrophy and filter feeding emerging as secondary strategies. However, higher δ^13^C values were measured in larger mussels, suggesting that they rely more heavily on carbon from methanotrophic endosymbionts as they grow (De Busserolles et al., 2009).

**Figure 4:**
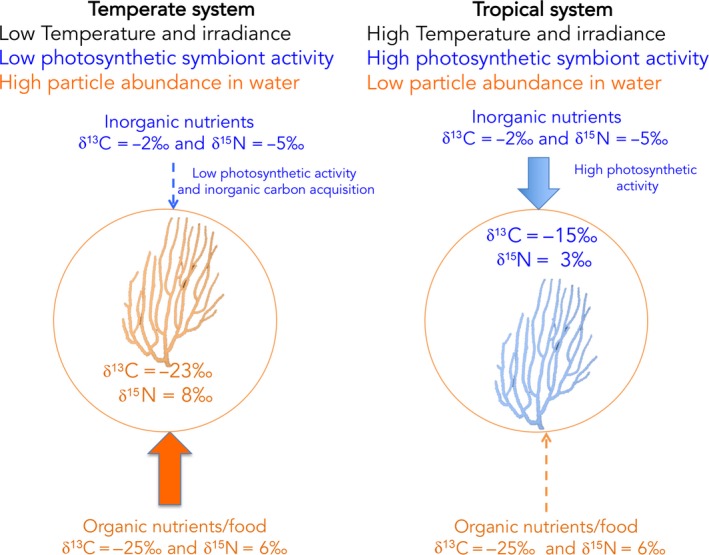
Isotopic variability in a temperate and tropical autotrophic symbiosis (dinoflagellate–gorgonian association). The isotopic signature of temperate organisms that live in a particle‐rich environment, and in which symbionts are not very active, will mirror the isotopic signature of the organic nutrients. In contrast, the isotopic signature of tropical gorgonians that live in oligotrophic and particle‐poor environments will be influenced by the activity of the symbionts and by their uptake of dissolved inorganic nitrogen. Isotopic data for the gorgonian tissue and organic food are from Cocito et al. (2013), for the temperate system and from Ward‐Paige, Risk, and Sherwood (2005), for the tropical system. Isotopic data for the inorganic carbon are from Gillikin and Bouillon (2007) and those for inorganic nitrogen are from York, Tomasky, Valiela, and Repeta (2007)

Several other factors can confound the identification of the food sources in symbiotic associations, such as unknown fractionation factors, the impossibility to isolate the symbionts and determine their δ^13^C value, or the small carbon contribution of the symbionts to the nutrition of the symbiosis. This latter situation applies, for example, to the temperate gorgonian (*Eunicella singularis*) that lives in symbiosis with *Symbiodinium* dinoflagellates, whose photosynthesis (and inorganic nutrient acquisition) is low all year round due to low irradiance experienced in temperate environments (Cocito et al., 2013). This gorgonian mostly relies on the host feeding and therefore shows δ^13^C (and δ^15^N) values close to the zooplankton values all year round (−23‰ and 8‰ for δ^13^C and δ^15^N, respectively), despite nutrient acquisition by the symbionts (Figure 4). Although autotrophically acquired nutrients from inorganic sources are expected to be transferred between the symbionts and the host, this nutrient source may represent only a small fraction of the total ingested nutrients and cannot be traced using the natural isotopic values. The reverse is obtained with shallow water tropical scleractinian corals (Figure 4). These organisms have few planktonic prey in the water and have to derive most of their carbon from the symbiotic dinoflagellates (Muscatine, McCloskey, & Marian, 1981). The δ^13^C values of the coral tissue are similar to those of the symbionts, and any heterotrophic input from the host will be masked by the large autotrophic input from the symbionts (Baker et al., 2015; Leal, Rocha, Anaya‐Rojas, Cruz, & Ferrier‐Pagès, 2017; Nahon et al., 2013; Reynaud et al., 2002). Finally, in autotrophic symbioses, the δ^13^C signature of the symbiotic association also largely varies according to the photosynthetic rates of the symbionts, which is primarily associated with light irradiance: Carbon fractionation increases with decreasing irradiance, which leads to a lower δ^13^C signature under low light (Heikoop et al., 2000). In this latter condition, δ^13^C of the symbiotic partners will reach the values of living or detrital particulate organic matter (POM) suspended in the water column, which makes it difficult to decipher if the predominant carbon source is autotrophic or heterotrophic. This is the case of scleractinian corals, which can thrive from 5 m, where light reaches a daily mean of 500 µmol photons m^−2^ s^−1^, down to 150 m depth, with an irradiance of 20 µmol photons m^−2^ s^−1^. In shallow waters, the δ^13^C of the coral tissue is significantly more positive (−10‰ to −14‰) than that of POM (ca. −20‰) (Muscatine, Goiran, Land, & Jaubert, 2005; Swart, Saied, & Lam, 2005), which clearly indicates that the symbiont photosynthates are the main carbon source of the coral tissue. With increasing depth, the δ^13^C of coral tissues becomes more negative (−23‰) and approaches that of the zooplankton (Swart et al., 2005). In this case, δ^13^C depletion can be due to a higher heterotrophic input following zooplankton ingestion (Muscatine, Porter, & Kaplan, 1989) or to a higher carbon fractionation by the symbionts with decreasing light (Swart, 1983; Williams, Röttger, Schmaljohann, & Keigwin, 1981), as well as higher internal carbon cycling between the host and the symbionts (Einbinder et al., 2009). The δ^13^C of a symbiotic association finally varies with the δ^13^C values of the seawater inorganic carbon sources. For example, when seawater has a high partial pressure in carbon dioxide (pCO_2_), such as in pCO_2_ vents, the δ^13^C values of the symbionts and the host can be significantly lighter than the signatures obtained under normal pCO_2_ levels (Horwitz, Borell, Yam, Shemesh, & Fine, 2015). Thus, in the symbiotic sea anemone *Anemonia viridis*, the δ^13^C value of symbionts shifted from −15‰ in control sites to −18‰ in the pCO_2 _vent, wrongly suggesting that the sea anemones were more heterotrophic in the vents.

One way to estimate the contribution of symbionts to the diet of their host is to calculate the difference between the δ^13^C of the host (δ^13^C_H_) and of the symbionts (δ^13^C_S_), also named *∆*δ^13^C_host‐symbiont_. The contribution of the symbionts to the diet of their host is inversely proportional to the *∆*δ^13^C_host‐symbiont_, or, in other words, the contribution will decrease from 100% to lower values when *∆*δ^13^C_host‐symbiont_ increases from zero to higher values. For example, low *∆*δ^13^C_host‐symbiont_ was recorded in coral species from surface waters of Moorea Island (from −1.44 ± 0.23‰ to 2.98 ± 0.58‰), which are corals that entirely rely on their symbionts for their energetic needs (Nahon et al., 2013). In opposite, the *∆*δ^13^C_host‐symbiont_ of deep corals relying on heterotrophic food sources could reach 8‰ (Muscatine et al., 1989).

As only one stable isotope does not always allow determining the main food source in a mixed diet, most studies often combine several stable isotopes (Phillips, 2012). Particularly, the δ^15^N value is often combined with δ^13^C to obtain a better identification of the main food sources or to better address the nutrient exchanges between the symbionts and their host.

### 3.2 δ^15^N** isotopes**

**Figure 5:**
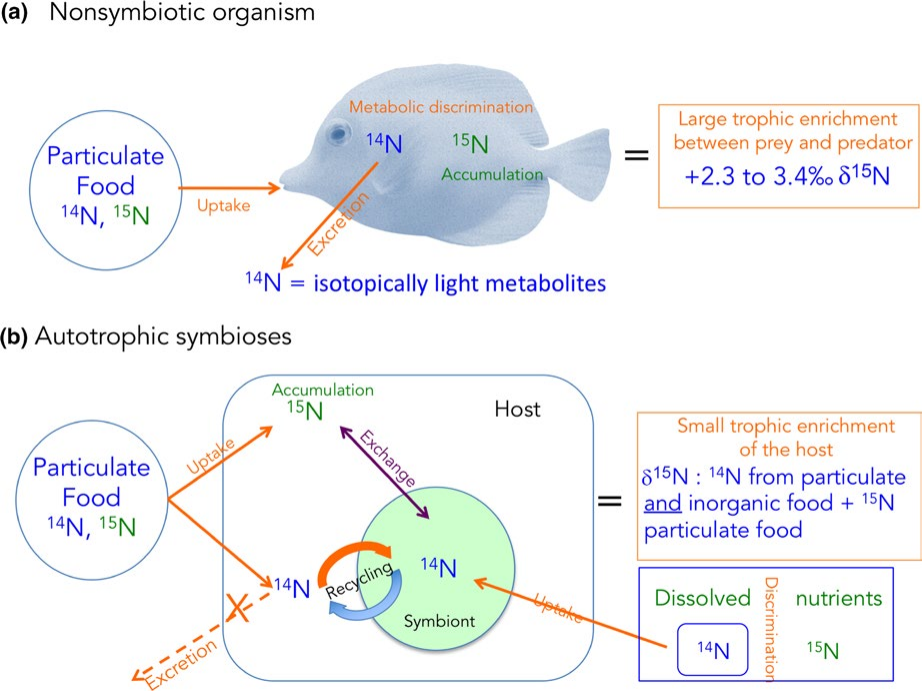
Difference in the isotopic signature of non‐symbiotic and symbiotic organisms. In non‐symbiotic organisms, the light isotopes are removed first during the processes of respiration and excretion, leaving the heavy isotopes in the tissue. The tissue will thus be enriched by ca. 3‰ in ^15^N and 1‰ in ^13^C at each trophic step. In symbiotic associations, nutrients are continuously recycled within the association, and the isotopic signature of the symbiosis will be a mix between the different food sources

The δ^15^N value of plants and animals varies according to two processes: assimilative and metabolic fractionation (Zanden & Rasmussen, 2001). Assimilative fractionation results from isotopic discrimination during nitrogen assimilation or isotopic differences between nitrogen pools. Metabolic discrimination is due to fractionation during amino acid transamination and deamination. During these processes in non‐symbiotic organisms, ^14^N amine groups are preferentially removed to produce isotopically light metabolites (Figure 5a), leaving the remaining nitrogen pool enriched in ^15^N (Gannes, O'Brien, & Del Rio, 1997). This metabolic fractionation induces an increase in the δ^15^N value between prey and predators (Figure 5a), which ultimately allows estimating the trophic position of consumers (Zanden & Rasmussen, 1999, 2001 ). It is often assumed that the δ^15^N value of a consumer is enriched by 2.3‰–3.4‰ over that of its diet (Minagawa & Wada, 1984), but the fractionation factor is species‐specific, and can differ significantly from this value (discussed in Zanden & Rasmussen, 2001). δ^15^N fractionation in carnivores is indeed relatively stable and varies within a narrow range (mean of 3.2‰ ± 0.4‰), whereas δ^15^N fractionation between plants and herbivores is highly variable (mean 2.5‰ ± 2.5‰; Zanden & Rasmussen, 2001). No study has, however, determined fractionation factors for symbiotic associations such as corals, probably because of the difficulties described below.

In symbiotic organisms, the δ^15^N value of the host (consumer) does not follow the same rule as for asymbiotic organisms. Due to the assimilative fractionation, the δ^15^N of the “entire” symbiotic association (i.e., host and symbionts) varies depending on the main nitrogen source(s) (atmospheric, dissolved, or particulate) used by each partner. The δ^15^N value also varies with the importance of the internal recycling between the symbionts and the host. Figure 5b is an example of the differences between symbiotic and asymbiotic organisms due to the internal recycling. Compared to asymbiotic organisms, the ^14^N of the particulate food ingested by the host is not excreted as waste product, but recycled by the symbiont, and re‐transferred to the host, thereby “diluting” the heterotrophic ^15^N enrichment. This host‐mediated signal is also combined with the symbiont‐mediated signal. Symbionts tend to take up the light isotope (^14^N) from the inorganic nutrient pool, leaving the heavy isotope (^15^N) in seawater. This light isotope is again translocated to the host for its own needs. The final δ^15^N value of the symbiotic association will thus be a mix of all these nitrogen sources. In addition, symbionts and hosts can assimilate nitrogen sources with different δ^15^N values (summarized in Sigman, Karsh, & Casciotti, 2009). In summary, atmospheric nitrogen (N_2_), which has a very low δ^15^N value close to 0‰ (Petersen et al., 2017), can only be fixed by diazotroph symbionts, which lowers the δ^15^N value of the symbiotic association (France, Holmquist, Chandler, & Cattaneo, 1998). Dissolved inorganic nitrogen sources (DIN), such as ammonium, nitrate, and urea, which are assimilated by the symbionts, have a higher δ^15^N, closer to 4‰–6‰. This is the same for the dissolved organic sources, taken up by both symbionts and hosts ((Sigman et al., 2009). Finally, particulate detrital or living nitrogen sources (PON) taken up by animal hosts display an even higher δ^15^N value (6‰‐14‰), except when it originates from mangrove forests, where the values are lower (Corbisier et al., 2006; Kao, Tsai, Shih, Tsai, & Handley, 2002; Riera, Stal, & Nieuwenhuize, 2004). In symbiotic associations, these different sources are often used in combination and continuously recycled within the association (Figure 5b), which make it difficult to interpret the δ^15^N value and the original nitrogen source. In autotrophic symbioses, for example, the δ^15^N value is often a mix between DIN taken up by the symbionts (Grover, Maguer, Reynaud‐Vaganay, & Ferrier‐Pages, 2002) and PON captured by the host (Houlbrèque & Ferrier‐Pagès, 2009), and is usually in the range of that of surface organic material (4‰–10‰). It is, therefore, difficult to estimate which nitrogen source is predominant. In addition, photosynthetic processes affect the final δ^15^N value of the symbiotic association. As for carbon, decreasing light level significantly decreases the δ^15^N value of the symbiotic association (Baker, Kim, Andras, & Sparks, 2011; Heikoop et al., 1998). This is due to an important assimilation of DIN by the symbionts under high light, which strongly depletes the host DIN pool and leads to a reduced fractionation relative to external DIN. In symbiotic associations involving more than one symbiont type, such as those containing both autotrophic and diazotrophic symbionts (Bednarz et al., 2017; Mohamed, Colman, Tal, & Hill, 2008), the δ^15^N value will be the result of both symbiont contributions. If the nitrogen supply by diazotrophs largely exceeds that by the algae/animal, the δ^15^N values of the animal tissue will be very low. This has been observed in autotrophic–symbiotic sponges that presented δ^15^N values ranging from 0‰ to 4‰ (Freeman & Thacker, 2011; Mohamed et al., 2008). A similar depletion in ^15^N (low to negative δ^15^N values) has been observed in chemoautotrophic and methanotrophic symbioses, because of an important contribution of N_2_ (Lee & Childress, 1994; Petersen et al., 2017) and an important biomass of methane‐oxidizing bacteria highly depleted in ^15^N (Macko, Fogel, Hare, & Hoering, 1987). Finally, the δ^15^N value of the symbiosis will also vary with anthropogenic DIN pollution, with a consistent δ^15^N enrichment in symbionts of polluted areas compared to oligotrophic ones (Baker, Murdoch, Conti‐Jerpe, & Fogel, 2017; Wong, Duprey, & Baker, 2017).

### 3.3 Other stable isotopes

δ^34^S is not commonly used to study nutritional symbioses, although it can be useful to trace the production of sulfur compounds such as DMS(P) by symbionts. These compounds are involved in multiple physiological functions in algae and bacteria, such as osmoprotection (Motard‐Côté & Kiene, 2015), antioxidant defense (Sunda, Kieber, Kiene, & Huntsman, 2002), dissipation of excess energy (Stefels, 2000), among others. DMSP production has been monitored in symbiotic protists (Gutierrez‐Rodriguez et al., 2017) and anthozoans such as corals (Gardner, Raina, Ralph, & Petrou, 2017; Jones, Curran, Swan, & Deschaseaux, 2017). The interpretation of isotopic changes in δ^34^S during DMSP cleavage into DMS is, however, complex, because of different fractionation factors between taxonomically different groups. For example, δ^34^S‐DMSP and δ^34^S‐DMS values were similar in microbial assemblages of the Red Sea, while the δ^34^S value of DMS produced by symbiotic acantharians was 1.5% lower than that of DMSP (Amrani, Said‐Ahmad, Shaked, & Kiene, 2013). In the same way, there is usually a slight ^34^S‐depletion in DMSP in symbiotic organisms compared to seawater SO_4_^2‐^, even though a high enrichment was also observed in a protist–microalgae symbiosis (Gutierrez‐Rodriguez et al., 2017). δ^34^S can also be used to help discriminate organic matter sources, particularly between terrestrial and marine sources (Granek, Compton, & Phillips, 2009), as well as in hydrothermal systems where sulfur is critical for biogeochemical cycles (Kennicutt II et al., 1992). Overall, these few studies with, sometimes, opposite results clearly show that more investigations are needed to understand the factors and processes affecting the δ^34^S value of symbiotic associations.

Future research involving stable isotopes in natural concentrations should start focusing on combining the isotopes reviewed above with others that have been ignored in marine nutritional studies. For example, changes in the stable isotope value of metals, such as δ^66^Zn and δ^65^Cu, have been widely used in mice and humans, to trace pathological conditions (Balter et al., 2015) or different dietary conditions (Costas‐Rodríguez, Van Heghe, & Vanhaecke, 2014; Jaouen, Pons, & Balter, 2013; Jaouen, Szpak, & Richards, 2016). In corals, δ^66^Zn and δ^65^Cu can also be used as tracers to record bleaching (Ferrier‐Pagès, Sauzéat, & Balter, 2018). Their combination with δ^13^C and δ^15^N, whose changes with environmental conditions or trophic status are better explored, is likely to bring novel insights into the nutritional interactions of marine symbioses.

### 3.4 Mixing models

The estimation of the contribution of the different food sources to a consumer's diet has largely benefited from Bayesian mixing models (Parnell et al., 2013; Phillips, 2012). These models take into account of the isotopic ratios of the consumers’ tissues and food sources, as well as the isotopic fractionation, or trophic enrichment factor, from the prey to the predator. Mixing models are, however, not always the best tools for tracing food sources, especially when the signature of the food sources largely overlaps in isotope space or when the consumers feed on a large diversity of prey. There are also other common problems that often lead to an incorrect use of mixing models. For instance, the type of food consumed by the animal host is often unknown, which can lead to the analysis of food sources that are ecologically irrelevant but that might show a stable isotope signature that fits within the stable isotopic niche potentially consumed by the target species.

The application of mixing models to marine mutualistic symbioses is relatively scarce, with most studies targeting food web dynamics of deep‐sea benthic communities. Levin and Michener (2002) were among the firsts using mixing models to show that the combination of low δ^15^N and δ^13^C values evidenced chemoautotrophic symbioses in bivalve and pogonophoran taxa. Using similar statistical methods, McLeod, Wing, and Skilton (2010) as well as Riekenberg, Carney, and Fry (2016) assessed the carbon contribution from chemoautotrophic and methanotrophic symbiotic bacteria to bivalve nutrition. In tropical symbioses, a mixing model was used by Freeman and Thacker (2011) to quantify the percentage of symbiont‐derived versus POM‐derived carbon assimilated by reef sponges with microbial symbionts.

While stable isotope mixing models are becoming increasingly used in ecology and evolution studies, as well as becoming more complex and accurate (Phillips et al., 2014), their application to provide new insights on the nutritional ecology of marine mutualistic symbioses is still poorly explored. This is likely associated with the poor number of studies using combined stable isotope values of the symbiotic host and symbionts, as well as the relatively low sample size that is used in such studies. Some of these studies (Cocito et al., 2013; Ferrier‐Pagès et al., 2011; Leal et al., 2014) calculated the theoretical food source based on subtracting a trophic enrichment factor from the δ^13^C and δ^15^N values of the host organism (1‰ for δ^13^C, and 3.5‰ for δ^15^N). While this simple approach is scientifically valid, it fails to consider several factors that are examined in mixing models, particularly the isotopic variability of the different food sources, the variability of trophic enrichment factors between consumers and the different food sources, and concentration dependence means, that is, the estimated proportion of carbon and nitrogen in each food source (Parnell et al., 2013). Moreover, the spatial distance between the theoretical food source and the isotopic values of the different food sources cannot be statistically analyzed using the stable isotope value of the theoretical food source. A mixing model approach can provide statistically robust estimates and confidence intervals for the contribution of each food source to the nutrition of the coral host. Future studies that aim to apply such mixing models should use a robust number of replicate samples to maximize the statistical accuracy of the mixing models. Second, the TEF used to fuel the model should be obtained empirically and not based on previous estimates. Not only more accurate TEF are needed, but variability estimates are also important to improve the quality of the mixing model output. Third, and although it is not mandatory, the mixing model requires the proportion of carbon and nitrogen of each food source. This primary and fundamental information is not always available as most studies fail to perform such simple analyses. For instance, while the carbon and nitrogen content of live planktonic organisms is usually known, their proportion in an individual organism is usually unknown. Such gap of knowledge is important to address in order to improve the estimates of stable isotope mixing models.

### 3.5 Compound‐specific isotope analyses (CSIA)

Compound‐specific isotope analysis (CSIA) has been increasingly used in ecological studies as a new tool for analyzing natural food webs, especially coupled with bulk stable isotope analysis. Compared to SIA, which is based on the stable isotope value of the total tissues or that of total plankton cells, CSIA corresponds to the isotopic signature of the organic matter compounds, that is, fatty acids (FA) and amino acid (AA). CSIA is at the biochemical building‐block level and thus allows tracing the exchange of precise molecules in a food web (Evershed et al., 2007). In addition, the interpretation of CSIA requires fewer assumptions than bulk isotopic values. Specifically, the physiological forces affecting isotopic values of a single group of compounds are less numerous and often better understood than the diversity of forces that are known to affect bulk tissues. CSIA is, therefore, particularly more successful than the other trophic markers when (a) organisms cannot be physically isolated from each other, such as in symbiotic associations; (b) when there is a need to trace quantitatively minor but qualitatively important components; or (c) when different food sources have similar bulk δ^13^C signatures (Gladyshev, Sushchik, Kalachova, & Makhutova, 2012).

**Figure 6:**
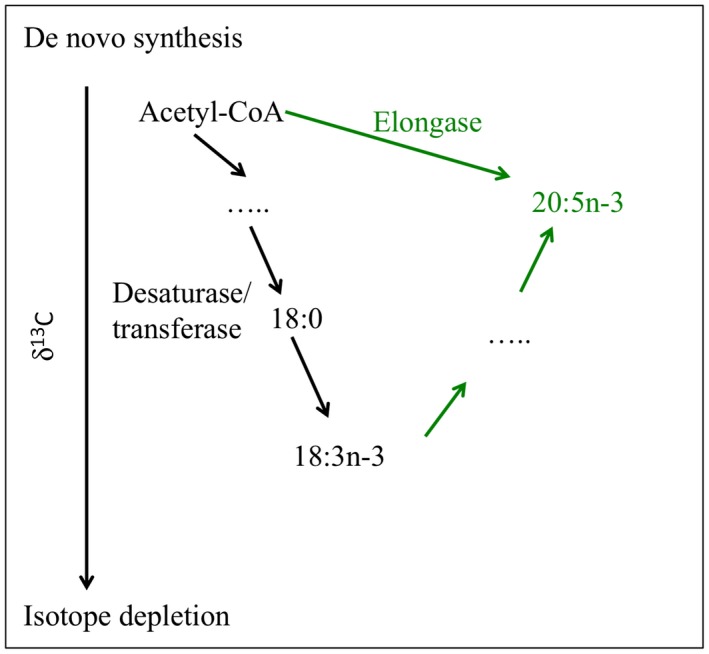
The δ^13^C of essential fatty acids (EFA) in the animal is comparable to that of the symbionts or the external prey, since little or no isotopic fractionation occurs during this process. However, for the de novo synthesis of fatty acids from pool of acetate in the animal tissue: Transferases/desaturases induce an isotope depletion in the synthesized fatty acid, while elongase adds carbon atom from the acetate pool and thereby provides the isotope enrichment in the synthesized fatty acid (modified from Gladyshev et al., 2012)

Fatty acid compound‐specific isotope analysis (CSIA‐FA) is based on the fact that δ^13^C values of a specific FA compound reflect its synthetic pathway and hence its source (Hayes, 1993; Teece, Fogel, Dollhopf, & Nealson, 1999). These values will thus be different when compounds are derived from direct biosynthesis or from an indirect dietary source (Abrajano Jr, Murphy, Fang, Comet, & Brooks, 1994; Fang et al., 1993). Essential fatty acids (EFA), such as the “omega‐6” and “omega‐3” FA (linoleic acid, arachidonic acid, eicosapentaenoic acid, and docosahexaenoic acid), cannot be directly synthesized by animals and have to be acquired through predation or symbiont transfer; therefore, the δ^13^C‐EFA values of the animal will be comparable to that of the symbionts or the external prey, since little or no isotopic fractionation occurs during this process (Treignier, Tolosa, Grover, Reynaud, & Ferrier‐Pagès, 2009). In the case of non‐essential FAs, which can be synthesized de novo by the animal, the δ^13^C values will reflect the competing processes of assimilation from external food and de novo synthesis (Gladyshev et al., 2012; Villinski, Hayes, Villinski, Brassell, & Raff, 2004). For the de novo synthesis of fatty acids, transferase and desaturase induce a δ^13^C isotope depletion in the synthesized fatty acid, while elongase provide a δ^13^C enrichment in the synthesized fatty acid (Figure 6). CSIA‐FA has been used in symbiotic associations for the first time to evaluate the dietary strategies of marine mytilids from a normal coastal ecosystem and from a cold hydrocarbon seep ecosystem (Abrajano Jr et al., 1994). Data showed that fatty acid δ^13^C compositions were more negative (depleted) for the seep mussels (−56.9‰ to −49.0‰ against −34.4‰ to −24.9‰), which relied almost exclusively on endosymbiotic methanotrophic bacteria as a carbon source, than for coastal mussels, which had a mixed dietary intake from a large diversity of food sources. After this pioneer work, CSIA‐FA has been extensively used to study nutritional pathways of chemosynthetic symbioses (Fang et al., 1993; MacAvoy, Macko, & Joye, 2002; Pruski et al., 2017; Streit, Bennett, Dover, & Coleman, 2015). For other symbiotic associations, FA‐CSIA has proved to be particularly useful in elucidating dietary preferences of scleractinian corals. The coral animal can indeed acquire essential FA directly, through the translocation of FA by their symbionts, or indirectly, through zooplankton predation. As δ^13^C values of FA produced by dinoflagellates and zooplankton differ by more than 5‰, FA δ^13^C of the coral tissue allows tracing which FA is obtained through one or the other pathway (Teece, Estes, Gelsleichter, & Lirman, 2011; Treignier et al., 2009). CSIA‐FA was also used to detect differences in feeding behavior within and between coral species and within reef sites (Teece et al., 2011).

**Figure 7:**
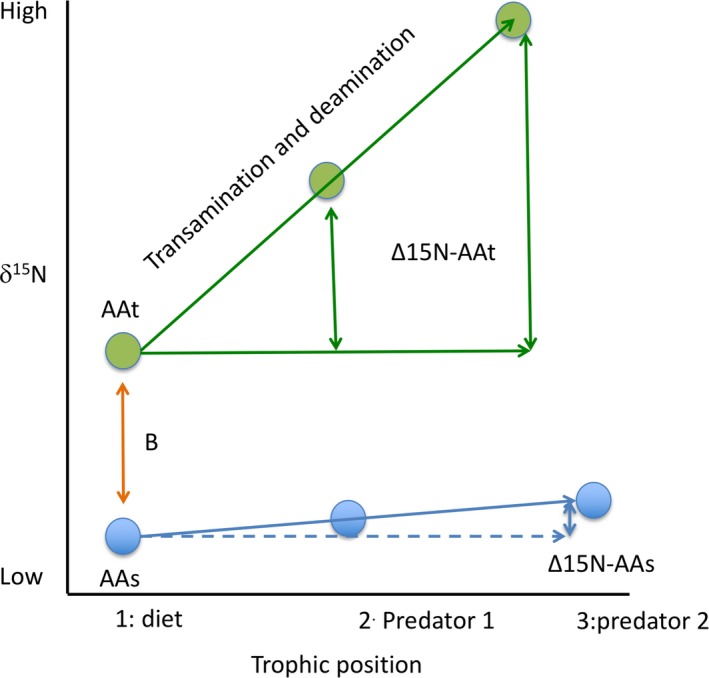
A schematic diagram of fractionation in individual amino acids nitrogen isotope value with trophic transfer. Trophic amino acids (AAt), with higher δ^15^N (B) than source amino acids (AAs), show large fractionation with each trophic transfer. On the contrary, source amino acids (AAs) show little to no fractionation with trophic transfer (adapted from McMahon et al., 2015)

Amino acid compound‐specific isotope analysis (AA‐CSIA, Popp et al., 2007) is based on the same principle as for FA, but has been much less developed for both normal trophic chains and symbiotic associations. Both carbon and nitrogen isotopes of AA can be used to understand diet composition and metabolic processes. The δ^15^N and δ^13^C values of the average amino acids often mirror bulk δ^15^N and δ^13^C (Boecklen et al., 2011; McClelland & Montoya, 2002). However, CSIA‐AA allows comparing “trophic” AAs with “source” AAs (Gerringer, Popp, Linley, Jamieson, & Drazen, 2017; McMahon, Hamady, Thorrold, & Review, 2013; McMahon, Thorrold, Elsdon, & McCarthy, 2015). Trophic AAs (AAt), mainly glutamic acid, alanine, aspartic acid, leucine, isoleucine, and proline, are strongly fractionated relative to diet (up to 7‰ for some AAs) during transamination and deamination, since they reflect variation in catabolism and origins of the carboxyl and amine groups (Figure 7). On the contrary, source or essential AAs (AAs), such as glycine, phenylalanine, and histidine, are not strongly fractionated relative to diet, because its dominant metabolic processing does not form or break C‐N bonds. δ^15^N and δ^13^C values of source AAs can thus provide information about the origin of nitrogen in an animal's food (Gerringer et al., 2017). To our best knowledge, CSIA‐AA has been used only once in a marine symbiotic association (Mueller, Larsson, Veuger, Middelburg, & Oevelen, 2014).

There are, however, limitations to the CSIA technique. At this time, only a limited number of laboratories provide CSIA services. Collaboration with academic laboratories is thus needed. In addition, while the method is quite sensitive, there are limitations for such sensitivity. For example, not all AA or FA can be obtained in sufficient quantity for the determination of their δ^13^C values (Tolosa, Treignier, Grover, & Ferrier‐Pages, 2011). Isotopic fractionation may also be so minimal that little or no isotopic enrichment is detected. This effect can occur in molecules with many of the same atoms (e.g., several C atoms in one molecule). Another problem is the variability in amino acid nitrogen isotope fractionation between diet and consumer across a wide range of species and diet types (McMahon et al., 2013). Finally, the samples cannot be derivatized and shipped for analysis, as they are suspended in volatile solvent and undergo loss. Future research should aim to develop a standard and easy procedure for these analyses in marine samples, so that more laboratories can apply CSIA.

## 4 ISOTOPIC LABELING EXPERIMENTS

The basic idea behind isotopic labeling experiments is that stable isotope‐enriched compounds (i.e., ^13^C, ^15^N, ^35^S‐compounds) have higher concentrations of the rare isotope than the natural abundance. The enrichment in such rare isotope can be used to follow its incorporation into specific molecules within an organism's tissue, exactly in the same way as for radioactive tracers. It can also be used to calculate incorporation and degradation rates, determine the transformation pathways, and identify which partner from the symbiotic association is involved in matter fluxes. The experiments consist in enriching seawater or particulate matter serving as a food source, and following the enrichment in the symbionts, the host, or surrounding medium during time‐series incubations. Both inorganic and organic compounds have been applied to symbiotic associations to measure the assimilation of different nutrient sources, the nutrient transfer to the symbiotic partner and/or external organisms, and overall make important conclusions about symbioses and evolution.

Tracing inorganic labeled compounds allows estimating the contribution of the symbionts to the provision of food to the symbiotic association. For example, ^13^C‐bicarbonate has been intensively used to follow the assimilation and partitioning of autotrophic and chemosynthetic‐acquired carbon in sponges (Freeman, Baker, Easson, & Thacker, 2015), corals (Tremblay, Naumann, Sikorski, Grover, & Ferrier‐Pagès, 2012), or deep vent organisms (Riou et al., 2008). ^15^N‐nitrate and ^15^N‐ammonium tracers have instead shown that the symbionts of most symbiotic associations, from the deep ones with methanotrophic or chemosynthetic bacterial symbionts, to the shallower ones with algal symbionts, are able to assimilate inorganic nitrogen dissolved in seawater and translocate them to the host (Freeman et al., 2015; Grover et al., 2002; Grover, Maguer, Allemand, & Ferrier‐Pagès, 2003; Liao et al., 2014; Morganti, Coma, Yahel, & Ribes, 2017; Rädecker, Pogoreutz, Voolstra, Wiedenmann, & Wild, 2015). Finally, ^15^N‐N_2_ gas was used to show the assimilation of dinitrogen by diazotrophs associated with animals such as sponges and corals (Bednarz et al., 2017; Benavides et al., 2016; Ribes, Dziallas, Coma, & Riemann, 2015), or by the chemosynthetic symbionts of some marine invertebrates (Petersen et al., 2017). Tracing labeled dissolved or particulate organic matter (DOM and POM, respectively) allows estimating the contribution of the animal host to the provision of food to the symbiotic association. However, few studies have followed the fate of DOM and POM within a symbiotic association as compared to inorganic compounds (Hughes & Grottoli, 2013; Piniak et al., 2003; Tremblay et al., 2015). Overall, the assimilation of inorganic or organic compounds by the symbionts and hosts varies with the environment, and the organisms’ trophic state and genotype. Therefore, it was demonstrated that the acquisition of autotrophic carbon was not correlated to symbiont abundance, but rather to the amount of light received by the symbiotic association or with the ratio of gross productivity to respiration (Freeman, Thacker, Baker, & Fogel, 2013; Tremblay, Grover, Maguer, Hoogenboom, & Ferrier‐Pagès, 2014). Exposure to light is an important parameter for the uptake of inorganic nitrogen (Grover et al., 2002), which also increases with the availability of nitrogen in seawater or with the nitrogen starvation state of the association (Kopp et al., 2013; Tremblay et al., 2015). In corals, symbiont identity determines the rates of inorganic carbon and nitrogen assimilation (Baker, Andras, Jordán‐Garza, & Fogel, 2013; Ezzat, Towle, Irisson, Langdon, & Ferrier‐Pagès, 2016). In particular, symbionts of clade D, which are known to present a particular resistance to thermal stress, are less efficient in assimilating nutrients than clade C, which is more widely distributed among coral species but is not resistant to high temperatures (Baker et al., 2013).

**Figure 8:**
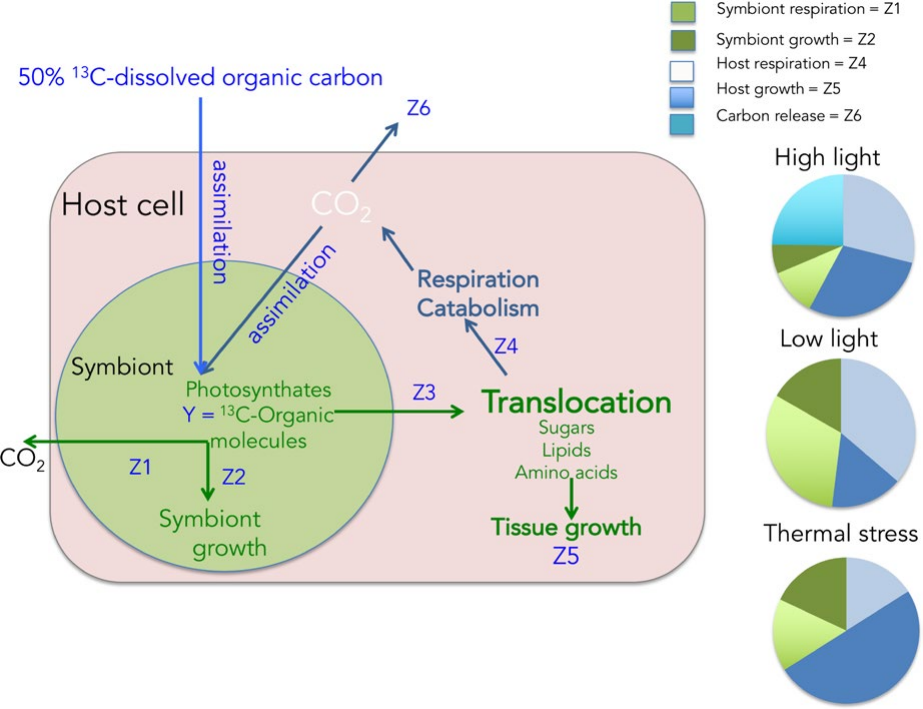
Autotrophic carbon budget in a coral–dinoflagellate symbiosis. The budget can be determined using ^13^C‐dissolved inorganic carbon (^13^C‐DIC), and measurements of gross photosynthesis and respiration of symbionts and host using oxygen measurements. The ^13^C can be followed within the symbiotic association, in particular the amounts retained in symbionts and host biomass. By applying adapted equations (Tremblay, Grover, Maguer, Legendre, & Ferrier‐Pages, 2012), the fate of the autotrophic carbon incorporated can be estimated under different conditions. The pie charts represent the percentage of the carbon incorporated into the host tissue and symbionts or lost after 24 hr under normal (high light), or under reduced light and high temperature conditions

Studies, which have followed the isotope tracer within the symbiotic association, have demonstrated that a fraction of the food assimilated by the symbionts or the host is translocated to the other partner for its own needs (Tremblay et al., 2015; Tremblay, Naumann, et al., 2012). Symbiont's food is also shared between symbiotic and non‐symbiotic tissues, as demonstrated in jellyfish, for which ^13^C‐labeled compounds are translocated from photosymbiont‐rich oral arm tissue to bell tissue (Freeman, Stoner, Easson, Matterson, & Baker, 2017). In corals, by coupling light and dark bottle incubations (P/R) with ^13^C‐bicarbonate tracers (Figure 8), it was shown that the percentage of autotrophic carbon assimilated by the symbionts (Z2 in Figure 8), translocated (Z3), and retained in the host tissue (Z5), or lost as respiration and mucus (Z6) depends on the environment and trophic state of the symbiotic association (Baker et al., 2015, 2018 ; Tremblay et al., 2016). Therefore, under low light (i.e., limited autotrophic acquisition) or warm conditions, coral symbionts sequester more resources for their own growth, thus parasitizing their hosts (Figure 8, pie charts). In octocorals, experiments using ^13^C‐bicarbonate have highlighted a correlation between colony morphology, polyp size, and productivity showing that productivity and polyp size have strong phylogenetic signals (Baker et al., 2015). A higher productivity was thus obtained for colonies with high polyp surface area/volume ratio. These productive species also maintained specialized, obligate symbioses, and presented a higher resistance to coral bleaching. On the contrary, generalist and facultative associations, with lower productivity, were more sensitive to bleaching. Finally, recent studies using ^13^C‐bicarbonate tracer also allowed following the fate of autotrophically acquired carbon by coral symbionts within the coral reef food chain (Rix et al., 2017, 2018, 2016 ). They labeled coral mucus with ^13^C and ^15^N and showed a transfer of mucus compounds into the tissue and phospholipid fatty acids of different sponge species living in tropical and cold water reefs environments, demonstrating a direct trophic link between corals and reef sponges. Part of the carbon and nitrogen transferred was subsequently released as detritus, feeding the sponge loop (Rix et al., 2016).

The use of enriched compounds in symbiotic associations has also several limitations. The first difficulty is to obtain a good separation of the host and the symbionts to accurately estimate the percentage of nutrients assimilated by each partner. In addition, the determination of assimilation rates and the calculation of fluxes (i.e., amount of nutrients transferred from one partner to the other one) require knowing the size of each compartment. Symbionts are generally representing a smaller fraction of the total biomass of the symbiotic association compared to that of the host. They are, therefore, more enriched in the labeled compound that the host (in terms of % total enrichment) since the assimilated compound is “diluted” in a smaller amount of biomass. However, when the “dilution factor” is taken into account, the assimilation rates can be equivalent. The same problem applies to the calculation of fluxes, which has to take into account the volume in which the labeled compounds is transferred. To avoid dilution problems, or to get a finer localization and identification of the compounds synthesized by each symbiotic partner, isotopes can be combined with different advanced technologies. For example, ^13^C‐bicarbonate has been combined with HPLC‐MS (high‐performance liquid chromatography, mass spectrometry) to investigate the lipogenesis in symbiotic cnidarians (Dunn, Pernice, Green, Hoegh‐Guldberg, & Dove, 2012). Results showed that fatty acids derived from the symbionts were not used directly in host lipogenesis, suggesting that additional sources of carbon, such as host heterotrophy could be important for the lipogenesis of FA in the host. Isotopes can also be combined with metabolomics instead of HPLC‐MS to trace each metabolite synthesized by both partners (see the review by Gordon & Leggat, 2010). For specific compounds, the relative metabolic contribution of different symbiotic partners can be assessed nowadays with single‐cell resolution, by combining isotopic enrichments with nanoscale secondary ion mass spectrometry (NanoSIMS) or matrix‐assisted laser desorption ionization mass spectrometry imaging (MALDI‐MSI; Kopp et al., 2015; Pernice et al., 2012). NanoSIMS can indeed provide direct imaging and quantification of seven different isotopes at the individual cell level (Achlatis et al., 2018; Berry et al., 2013; Kopp et al., 2013; Musat, Foster, Vagner, Adam, & Kuypers, 2012; Pernice et al., 2012). Although NanoSIMS has a great potential yet to be explored, it currently has numerous limitations, such as a limited number of reference materials, a reduced number of research facilities were able to afford this expensive equipment, and only a few of those have an imaging interface for live tissues; NanoSIMS has been primarily used for geological and cosmochemical applications. MALDI‐MSI rather enables identification of compounds on surfaces, such as in tissue sections (Gagnon et al., 2012). For instance, it has been used to localize metabolites within the tissues (epidermis, gastrodermis) of symbiotic and asymbiotic sea anemones (Kopp et al., 2015).

## 5 CONCLUSIONS

In moving forward toward understanding nutritional interactions in marine symbiotic associations via stable isotope analysis, a better understanding of the sources and processes affecting nutrient isotopes in each environment and within each symbiotic association is urgently needed. Indeed, the isotopic values of the different food sources, especially of the dissolved organic and inorganic sources, are not always precisely known, and the fractionation factors corresponding to the assimilation and exchanges of nutrients are even less understood. In addition, in natural abundance studies, multiple isotope markers (^13^C, ^15^N, ^34^S) have to be taken into account to constrain organic matter sources used by the symbiotic associations. Furthermore, analyzing the nitrogen and carbon stable isotope content of specific amino acids and fatty acids could be very useful, since some compounds show no change with trophic level, and therefore could be used to indicate the isotopic composition of the nutritional sources. Simultaneously, other compounds show a large increase with each trophic level, thereby allowing for definition of trophic level. Such an analysis could also help to better calibrate the bulk tissue isotope values. For such compound‐specific isotope analyses, the technique of HPLC‐IRMS would greatly broaden the types of biomarkers that can be analyzed. Several attempts have been published to directly couple HPLC with high precision isotope analysis. Commercial machines are however not available and sensitivity in terms of amounts of carbon needed is still rather low, so further development of these machines is needed. Finally, it is expected that future experimental manipulations using compounds enriched in δ^13^C and δ^15^N, followed by cell separation and compound‐specific isotope analyses, will shed considerable light on both symbiont and host metabolic pathways.

## CONFLICT OF INTEREST

The authors have no conflict of interests.

## AUTHORS’ CONTRIBUTIONS

Both authors equally contribute to this manuscript, from generating to the idea to writing the manuscript.

## DATA ACCESSIBILITY

No data were used in this manuscript.
