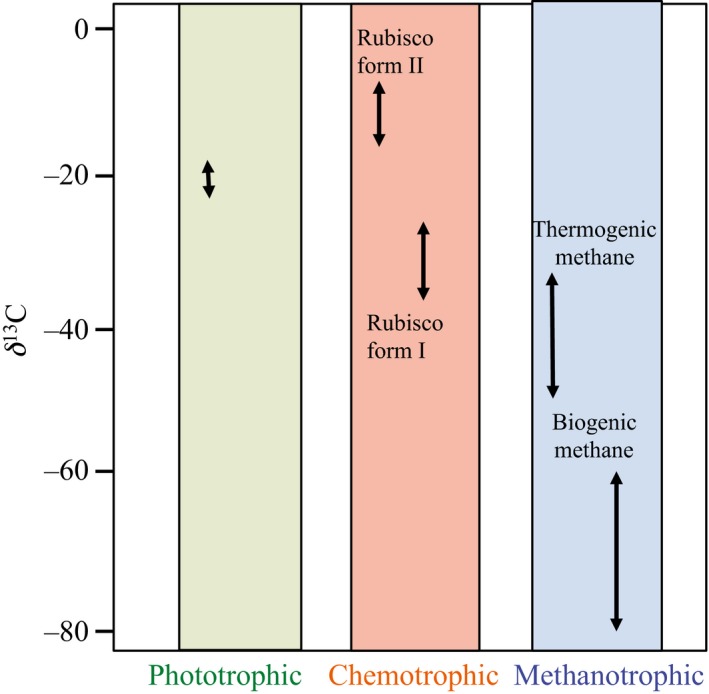# Corrigendum

**DOI:** 10.1002/ece3.5362

**Published:** 2019-06-27

In “Stable isotopes as tracers of trophic interactions in marine mutualistic symbioses,” which was published in issue 1, January 2019, Figure 3 is incorrect. The correct Figure 3 is printed below.